# Global Path Planning for Articulated Steering Tractor Based on Multi-Objective Hybrid Algorithm

**DOI:** 10.3390/s24154832

**Published:** 2024-07-25

**Authors:** Ning Xu, Zhihe Li, Na Guo, Te Wang, Aijuan Li, Yumin Song

**Affiliations:** 1 College of Agricultural Engineering and Food Science, Shandong University of Technology, Zibo 255000, China; xuning@saas.ac.cn (N.X.); 22503030106@stumail.sdut.edu.cn (N.G.); 2 Shandong Academy of Agricultural Machinery Sciences, Jinan 252100, China; 3 Huang Huai Hai Key Laboratory of Modern Agricultural Equipment, Ministry of Agriculture and Rural Affairs, Jinan 250100, China; 4 School of Automotive Engineering, Shandong Jiaotong University, Jinan 252100, China; 23104022@stu.sdjtu.edu.cn (T.W.); liaijuan@sdjtu.edu.cn (A.L.); songyumin@sdjtu.edu.cn (Y.S.)

**Keywords:** articulated steering tractor, differential evolution genetic algorithm, hybrid path-planning algorithm

## Abstract

With the development of smart agriculture, autopilot technology is being used more and more widely in agriculture. Because most of the current global path planning only considers the shortest path, it is difficult to meet the articulated steering tractor operation needs in the orchard environment and address other issues, so this paper proposes a hybrid algorithm of an improved bidirectional search A* algorithm and improved differential evolution genetic algorithm(AGADE). First, the integrated priority function and search method of the traditional A* algorithm are improved by adding weight influence to the integrated priority, and the search method is changed to a bidirectional search. Second, the genetic algorithm fitness function and search strategy are improved; the fitness function is set as the path tree row center offset factor; the smoothing factor and safety coefficient are set; and the search strategy adopts differential evolution for cross mutation. Finally, the shortest path obtained by the improved bidirectional search A* algorithm is used as the initial population of an improved differential evolution genetic algorithm, optimized iteratively, and the optimal path is obtained by adding kinematic constraints through a cubic B-spline curve smoothing path. The convergence of the AGADE hybrid algorithm and GA algorithm on four different maps, path length, and trajectory curve are compared and analyzed through simulation tests. The convergence speed of the AGADE hybrid algorithm on four different complexity maps is improved by 92.8%, 64.5%, 50.0%, and 71.2% respectively. The path length is slightly increased compared with the GA algorithm, but the path trajectory curve is located in the center of the tree row, with fewer turns, and it meets the articulated steering tractor operation needs in the orchard environment, proving that the improved hybrid algorithm is effective.

## 1. Introduction

The articulated steering tractor adopts an articulated steering mechanism. The whole machine has the characteristics of a small turning radius, compact structure, good passability, etc. [1], and it has the advantages of high operating efficiency and good driving performance when used in the orchard. Global path planning is one of the core tasks of path planning [2], which has an important impact on both the safe automatic driving of the articulated steering tractor and the actual work efficiency. Therefore, studying the global path-planning algorithm of articulated steering tractors is of great significance for the realization of intelligent and unmanned orchards in China. Global path planning in the orchard needs to be determined according to the actual operating results as well as the structure of the machine, such as spraying operations, with sprayer nozzles symmetrically distributed on both sides—spraying operations at the same time should ensure that the spraying effect on both sides of the rows of the tree is the same; weeding operations to achieve the elimination of weeds because the orchard rows of herbicide operations through the soil tilling will be covered by the weeds underground; open furrow fertilizer operations on both sides of the open furrow fertilizer machine in the middle of the tree rows for digging furrows to achieve the strip furrow fertilizer. According to the above orchard operations and the requirements of the machine structure, the path should be located in the middle of the tree rows. In addition, it is important to consider the accessibility of the tractor as well as comfort and, ultimately, determine the demand for the orchard operation where the path is located in the centerline of the tree rows, it is the shortest path, the path is smooth and turns in line with kinematic constraints [3], and so on. However, most of the current research is only carried out with the shortest path and smoothness as the goal, and the planned route cannot meet the actual operational needs of the orchard. At the same time, most of the research is to improve the algorithm itself, and a single algorithm in the face of a complex environment planning efficiency is low [4].

At present, path-planning algorithms mainly include the A* algorithm, ant colony optimization algorithm, dynamic window algorithm, genetic algorithm, simulated annealing algorithm, particle swarm optimization algorithm, and so on. The A* algorithm responds quickly to the environment and is the most effective direct search method in a static environment. The genetic algorithm has strong robustness and is suitable for solving complex optimization problems. However, the A* algorithm is not applicable to path-planning problems with multi-objective constraints. The traditional genetic algorithm has the disadvantages of slow convergence speed, poor local search ability, and poor quality of the solution. In search of improving the global path-planning algorithm, Liu et al. [5] proposed a hybrid heuristic function combining Euclidean distance and point–line distance, which reduces the number of search nodes of the A* algorithm, improves the search speed, and is suitable for path planning of indoor cleaning robot. Zhang et al. [6] introduced the artificial potential field into the heuristic function of the A* algorithm, used the diagonal distance method to calculate the estimated substitution price, and used the quartic spline b curve to smooth the path, which reduced the inflection point in the path planning and improved the work efficiency; however, the algorithm is complex, which increases the calculation time. Huang et al. [7] proposed a hybrid A* ant colony algorithm based on dynamic feedback, which uses a simplified A* algorithm to optimize the ant colony algorithm to solve the blindness of the initial search. Wang et al. [8] proposed a new adaptive genetic algorithm that designed the fitness function according to the path length and the number of inflection points, improved the roulette wheel selection method, and designed the adaptive crossover operator and mutation operator. The improved genetic algorithm shortened the length of the planned paths and reduced the number of inflection points in the paths and the number of iterations of the algorithm. Wahab et al. [9] improved the population initialization method of the genetic algorithm to provide stronger guidance in the initial solution construction process and proposed a new combination of genetic operators that gave the genetic algorithm better global and local search capabilities. Liang et al. [10] proposed an improved fusion ant colony and genetic algorithm for path planning of inspection robots, which improved the fitness function and a genetic operator of the genetic algorithm; the improved genetic algorithm together with B-spline optimized the path searched by the ant colony algorithm and obtained a smoothed path curve. Shi et al. [11] also integrated the improved genetic algorithm with the improved ant colony algorithm, which solved the problems of poor local search ability of the genetic algorithm and the tendency of the ant colony algorithm to fall into local optimization. Li et al. [12] introduced the population fitness variance, adaptive elite retention factor, and regeneration operator into the traditional genetic algorithm, adopted improved adaptive selection, crossover, and mutation probability, and increased the path turning angle and number of turning points in the fitness function. In addition, dynamic obstacle avoidance was realized by combining the improved dynamic window method. The above research mainly aims at the situation where the goal is the shortest path and the maximum smoothness and where there are great limitations for the applications in orchard operations. In the area of global path planning for agricultural machinery, Shen et al. [3] planned the path through the A* algorithm, projecting the feature points between rows to the centerline of the tree row to realize that the path was located on the centerline of the tree row and optimizing the trajectory by cubic nonuniform B-spline pairs; however, he did not consider the existence of obstacles in the centerline of the path.

Path tracking control is one of the key technologies to realize the automatic navigation of agricultural machines [13] according to the developed control algorithm to make the agricultural machine track the reference path within a certain accuracy range and follow the planned path. Fernando et al. [14] proposed a new multiple-input multiple-output (MIMO) linear variable parameter (LPV) controller, which takes into account the rolling dynamics of the vehicle and the onboard communication delays to improve the safety and comfort of an automated vehicle. He et al. [15] designed an unmanned path tracking control method based on model predictive control (MPC) for agricultural machines in paddy fields.

In order to solve the problem that most of the current global path planning does not meet the needs of orchard operations, this paper proposes a hybrid global path-planning algorithm based on the combination of an improved bidirectional search A* algorithm and an improved genetic algorithm. First, the bidirectional search improved A* algorithm is proposed to solve the problems of slower convergence and too many search nodes of the traditional A* algorithm. Second, by combining the differential evolution genetic algorithm, modifying the genetic algorithm fitness function and search strategy, and solving the problems of genetic algorithm path planning, it is easy to fall into the local optimum and path confusion where the path does not meet the actual orchard operation requirements. Finally, the global path is smoothed based on cubic spline curves, and the kinematic constraints of the articulated steering tractor are added to solve the problems in path planning such that the trajectories at the turns are not smooth and the curvature does not conform to the kinematic constraints of the articulated steering tractor.

## 2. Kinematic Modeling of Articulated Steering Tractor

**Figure 1:**
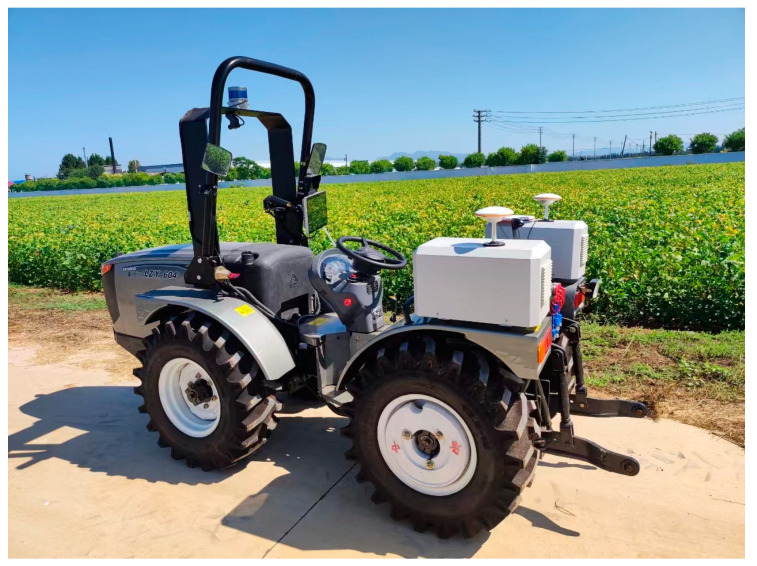
Complete structure of articulated steering tractor.

The steering mechanism of the articulated steering tractor is articulated, which ensures greater flexibility and reduces the turning radius relative to the general tractor, and the whole structure of the articulated steering tractor is shown in Figure 1.

**Figure 2:**
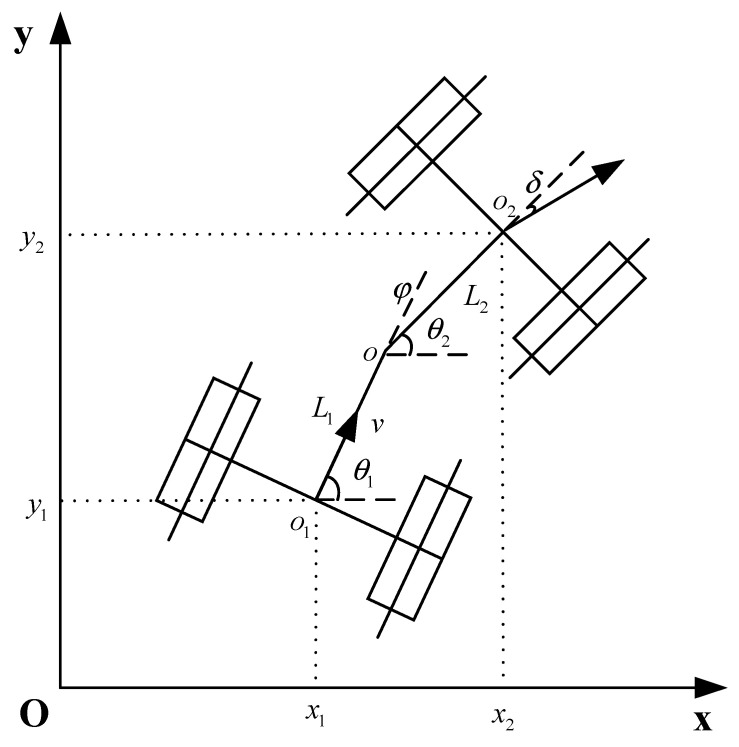
Kinematic model of articulated steering tractor chassis.

The articulated steering tractor travels slowly during operation and is subjected to less lateral force during low-speed traveling. Assuming that it does not experience a side-slip phenomenon, only the kinematic model of the articulated steering tractor is considered for path planning in this paper [16]. Obtaining the constraints during the motion of the articulated steering tractor described based on the kinematic model can make the planned path more reliable and realistic. The kinematic model of the articulated steering tractor is established based on the assumptions that the wheels of the articulated steering tractor are purely rolling, the front and rear bodies are, respectively, rigid bodies, and the rear wheels are driven [17], as shown in Figure 2.

In Figure 2, $\left(x_{1},y_{1}\right)$ is the coordinate of the center of the rear axle $O_{1}$, $\left(x_{2},\;y_{2}\right)$ is the coordinate of the center of the front axle $O_{2}$, $\theta_{1}$ is the azimuth of the rear tractor body, $\theta_{2}$ is the azimuth of the front tractor body, φ is the heading angle, δ is the corner of the front wheel, V is the central speed of the rear axle, $L_{1},\;L_{2}$ are the distance from the hinge point to the rear axle and the front axle, respectively. The kinematic model of the entire vehicle of the articulated steering tractor can be represented as:

(1) $$\left[\begin{array}{c} \begin{array}{c} \dot{x}\\ \dot{y} \end{array}\\ {\dot{\theta }}_{1}\\ \dot{\varphi } \end{array}\right]=\left[\begin{array}{c} \begin{array}{c} \cos\theta_{1}\\ \sin\theta_{1} \end{array}\\ \frac{\left(\sin\varphi -\tan\delta \cos\varphi \right)}{L_{1}\left(\cos\varphi +\tan\delta \sin\varphi \right)+L_{2}}\\ 0 \end{array}\begin{array}{c} \begin{array}{c} 0\\ 0 \end{array}\\ \frac{L_{2}}{L_{1}\left(\cos\varphi +\tan\delta \sin\varphi \right)+L_{2}}\\ 1 \end{array}\right]\left[\begin{array}{c} V\\ \omega \end{array}\right]$$

where $\dot{x},\dot{y}$ are the components of the velocity V on the x,y axes, respectively, ${\dot{\theta }}_{1}$ is the derivative of the rear body azimuth angle with respect to time, $\dot{\varphi }$ is the derivative of the heading angle with respect to time, and ω is the rate of change of heading angle.

The kinematic model of the articulated steering tractor describes the additional limits and constraints of the tractor motion that are not induced by forces [18] such as the rate of change of position equal to the velocity. Based on the above kinematic model, the orchard articulated steering tractor path planning needs to meet the following requirements:

(1) Articulated steering tractor turning radius constraint. In order to ensure that the planned path is practicable and avoid the problem that the tractor cannot be steered, the curvature of any point in the planned path should meet the minimum turning radius constraint.
(2) The constraint of curvature continuity of the articulated steering tractor. Unnecessary details and sharp corners in the planning path lead to sharp stops and turns during the operation of the articulated steering tractor, affecting the smoothness and comfort of the articulated steering tractor. The path is smoothed using cubic b-spline curves and the continuity at the nodes is $c^{2}$, which is sufficient to satisfy the curvature continuity.

## 3. Algorithm Description

### 3.1. A* Algorithm

**Figure 3:**
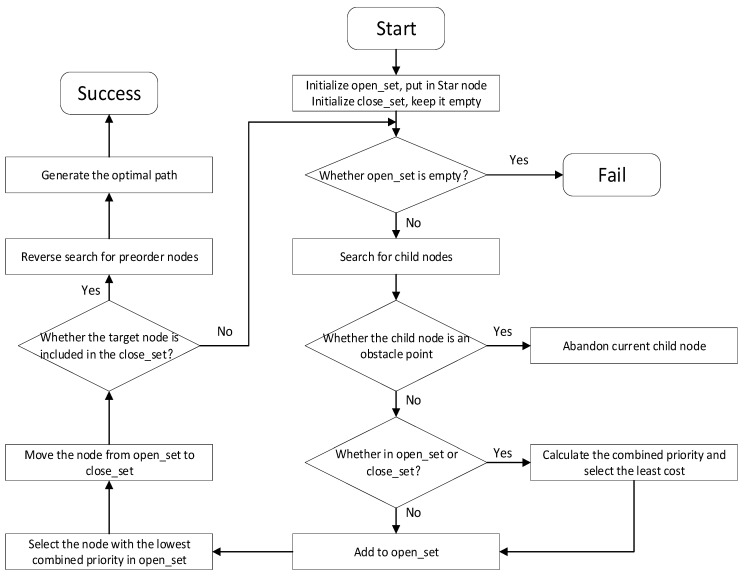
Flowchart of the A* algorithm.

The A* algorithm is a heuristic search algorithm that combines the breadth-first search (BFS) algorithm and Dijkstra algorithm [19], which changes the search performance by means of a heuristic function to find the shortest path faster, and it is the most efficient and direct search method for solving the shortest path in a static environment [20]. The specific flowchart is shown in Figure 3, and the integrated prioritization function is as follows:

(2) $$f\left(n\right)=g\left(n\right)+h\left(n\right)$$

where, n is the point to be searched, $f\left(n\right)$ is the integrated priority, $g\left(n\right)$ is the actual cost from the starting point to the current point, and $h\left(n\right)$ is the predicted cost from the current point to the end point.

### 3.2. Genetic Algorithm

**Figure 4:**
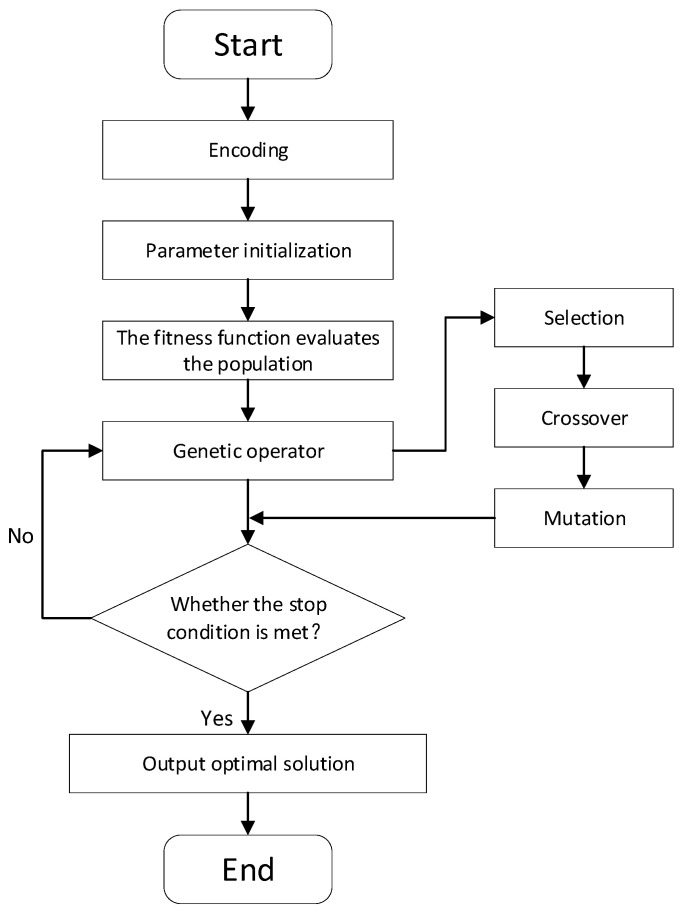
Flowchart of the genetic algorithm.

The genetic algorithm (GA) [21] is a typical heuristic algorithm, which has strong global optimization ability and is widely used in path planning. The specific flow of the genetic algorithm is shown in Figure 4. In the initial stage of the genetic algorithm, a random search is performed on the population, the fitness of the solutions is evaluated based on the search results—the higher the fitness, the stronger the probability of being selected in the tournament—and solutions with low fitness are not easily selected. The two selected solutions cross-mutate to form a new parent population and continue to iterate until the condition is satisfied [22]. Traditional genetic algorithms for path planning have some problems, such as a single initial population, the tendency to fall into local optimal solutions, and too many redundant points.

(1) Population initialization

Population initialization is particularly important in genetic algorithms for solving path-planning problems to ensure both path feasibility and path continuity (i.e., no jump walking).

Step 1: Randomly take a grid that is not an obstacle in each row of the raster map.

Step 2: To make the discontinuous paths of random search continuous, it is necessary to judge whether the adjacent sorted grids are continuous. The judgment method is as follows:

(3) $$D=max\left\{abs\left(x_{i}-x_{i+1}\right),abs\left(y_{i}-y_{i+1}\right)\right\}$$

where $\left(x_{i},y_{i}\right)$ and $\left(x_{i+1},y_{i+1}\right)$ are the coordinates of the two neighboring grids. If D = 1, it is continuous; otherwise, it is discontinuous. For discontinuous coordinates use the midpoint method to insert a new raster. The calculation is as follows:

(4) $$X_{new}=int\left(\frac{x_{i+1}+x_{i}}{2}\right)\;Y_{new}=int\left(\frac{y_{i+1}+y_{i}}{2}\right)$$

Step 3: Continue to determine whether the newly inserted grid and the previous grid are consecutive; cycle the above steps until the entire path is continuous.

(2) Fitness function

The fitness function is a criterion for distinguishing the goodness of individuals within a population based on a set objective function. The initial population is substituted into the fitness function and the fitness value is calculated to indicate the goodness of individuals [23]. If only the shortest path is considered, the expression of the fitness function is as follows:

(5) $$f=\frac{1}{length}$$

where length is the distance from the start to the end of the path.

(3) Selection

Tournament selection is used to simulate the competitive process of a tournament to build a new generation of populations by continuously selecting winners [24]. Each tournament selects a number of individuals for comparison; it selects the individual with higher (or lower) fitness as the winner and repeats the above steps until a sufficient number of winners have been selected. This process is deterministic and does not involve probability.

(4) Crossover

**Figure 5:**
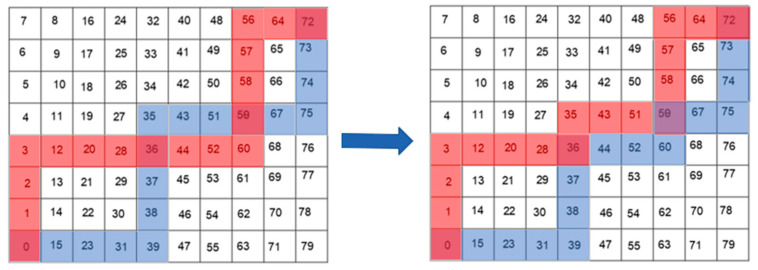
Genetic algorithm multipoint crossover process.

Figure 5 shows the genetic algorithm path-planning multipoint crossover process. Multipoint crossover refers to the randomization of multiple crossover points in individual coding strings, followed by partial gene exchange in the form of spaced exchanges [18]. The actual path planning is to find out all the same points in the two paths except the start and end points, randomly select more than one of these points, and intersect the spaced paths in a crossover operation.

(5) Mutation

**Figure 6:**
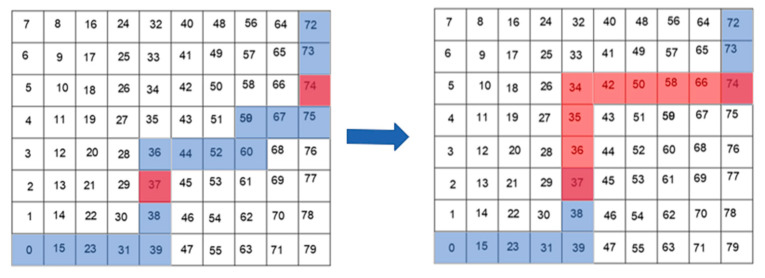
Genetic algorithm mutation process.

The mutation process is shown in Figure 6, which randomly selects two rasters in the path, removes the path between these two rasters, takes these two as neighboring rasters, and performs population initialization operations on these two points until a new continuous path is generated [25]. The mutation operation increases the global search capability of the algorithm to avoid falling into local optimal solutions.

### 3.3. Improved Hybrid A* and Differential Evolution Genetic Algorithm

In this paper, an improved A* algorithm with the differential evolution genetic hybrid algorithm (AGADE) is proposed. First, the A* algorithm and the traditional genetic algorithm (GA) are improved separately; then, the suboptimal solution obtained from the improved A* algorithm is used as the initial population of the improved genetic algorithm to realize the algorithm hybridization, which finally forms the AGADE hybrid algorithm.

#### 3.3.1. Improvement of the A* Algorithm

(1) Improvement of the integrated priority function

The integrated priority function directly affects the effectiveness of the A* algorithm, and this paper improves the integrated priority function of the A* algorithm:

(6) $$f\left(n\right)=g\left(n\right)+\omega \left(n\right)h\left(n\right)$$

Weights $\omega \left(n\right)$ are added to the combined priority of the A* algorithm. $\omega \left(n\right)$ affects the evaluation value, and the effect of $h\left(n\right)$ on the A* algorithm is changed by varying $\omega \left(n\right)$ [26]. The smaller $\omega \left(n\right)$ is, the more the A* algorithm tends toward the Dijkstra algorithm where the search speed is slow but the path is the shortest; the larger $\omega \left(n\right)$ is, the more the A* algorithm tends toward the BFS algorithm where the search speed becomes faster but the path is not guaranteed to be the shortest [27]. The weighting function $\omega \left(n\right)$ is expressed as follows:

(7) $$\omega (n)=\left\{\begin{array}{cc} \frac{d(h)}{d(g)}, & \frac{d(h)}{d(g)}\geq 1\\ 1 & \frac{d(h)}{d(g)}<1 \end{array}\right.$$

where $d\left(h\right)$ represents the Euclidean distance from the current node to the target point and $d\left(g\right)$ represents the Euclidean distance from the current node to the starting point.

(2) Improvement of the search method

Compared with the A* algorithm, the bidirectional A* algorithm searches from both the start and end points, which significantly reduces the number of nodes to be searched, especially in the graph search problem. When the size of the graph is large, the improvement of the search efficiency is more obvious [28]. The practical steps are as follows:

Step 1: Initialize the start and end points and assign them initial heuristic function values (i.e., estimate the distance from the start point to the end point), respectively.

Step 2: Initialize the start and end priority queues to hold the nodes to be expanded, respectively.

Step 3: Start searching from the start and end points simultaneously and select the node with the lowest priority for expansion each time.

Step 4: For the currently selected node, calculate and update its heuristic function value, generation value (i.e., the actual path length from the starting point to the node), and the total estimate (i.e., the heuristic function value plus the generation value).

Step 5: At each expansion of a node, check if any node has already been visited in the other direction and if there is a crossover node, find a path and return it.

Step 6: If there are no intersecting nodes, continue to select the next lowest priority node for expansion until the path is found or all nodes are searched.

Step 7: If no path is found after searching all the nodes, it means that there is no reachable path between the start point and the end point.

#### 3.3.2. Improvement of Genetic Algorithm

(1) Improvement of the fitness function

Path planning for an articulated steering tractor in the orchard environment should consider not only the path length but also the operational requirements, such as whether the path is located in the middle of the tree rows and whether the tractor should make frequent turns in practical applications, which increases the energy consumption [29]. Since this paper presents a hybrid algorithm, the path length problem is considered in the A* algorithm and is not repeated in the genetic algorithm, and the fitness function of the genetic algorithm [30] considers the middle of the path offset factor, the smoothing factor, and the safety factor. The new fitness function is as follows:

(8) $$fit=a\times fit_{1}+b\times fit_{2}+c\times fit_{3}$$

where a, b and c are weighting factors.

Distance constraints are added to the passable points to ensure that the points added during the iteration of the genetic algorithm are not too close to the obstacles.

The $fit_{1}$ is the tree row center offset factor of the path:

(9) $$fit_{1}=Xmin_{i}-d/2$$

where $Xmin_{i}$ is the minimum distance between the ith passable point and the obstacle and d is the orchard row spacing.

The articulated steering tractor should not change direction frequently and significantly during traveling as path smoothing can effectively reduce energy and time loss, and path smoothness is evaluated by path curvature. Using the three-point curvature method, three individuals $P_{i-1},P_{i},\;P_{i+1}$ are taken from the population. The $fit_{2}$ is the smoothing factor as follows:

(10) $$fit_{2}=\frac{2\sin B}{b}$$

where B is the angle between $P_{i-1}P_{i}$ and $P_{i}P_{i+1}$, b is the distance between $P_{i-1}$ and $P_{i+1}$.

The $fit_{3}$ is the safety coefficient [11]:

(11) $$fit_{3}=\overset{n-1}{\underset{i=1}{\sum }}\frac{1}{S_{i}}$$

where $S_{i}$ is the safety penalty value of the path point i. The safety distance of the point is measured by the presence of obstacles in the 8-grid neighborhood of the path node. If there is no obstacle grid in the 8 neighbors of a path node, the point is a safe movement point. Otherwise, the point is a safety hazard and the penalty value $S_{i}$ is added 1. The fewer the obstacles, the safer it is; the smaller $S_{i}$ is, the larger the safety factor will be and the larger the fitness function will be.

(2) Improvement of the search strategy

Genetic algorithms are prone to fall into local optimal solutions, especially in complex high-dimensional spaces [31], so this paper proposes a more diversified and effective genetic algorithm search strategy based on differential evolutionary algorithms to address the above problems.

On the basis of the genetic algorithm, differential evolution introduces new individuals and generates these new individuals by performing mutation and crossover operations on the current population of individuals, which increases the diversity of the algorithm, improves the algorithm’s ability to explore the solution space and the algorithm’s ability to search globally, and helps to find a better solution faster while avoiding falling into a local optimum solution. The mutation formula is as follows:

(12) $$v_{i}\left(g+1\right)=x_{r1}\left(g\right)+F\times \left(x_{r2}\left(g\right)-x_{r3}\left(g\right)\right)$$

where $x_{r1},x_{r2},x_{r3}\ldots x_{rn}$ are n individuals. F is the scaling factor 0.5. i denotes the ith individual, $v_{i}$ is the newly generated solution and g denotes the generation.

The crossover formula is as follows:

(13) $$v_{i,j}=\left\{\begin{array}{c} h_{i,j}\left(g\right),rand\left(0,1\right)\leq cr\\ x_{i,j}\left(g\right),else \end{array}\right.$$

where j denotes the jth dimension value of the ith individual, cr is the crossover probability, and $h_{i,j}\left(g\right)$ represents the jth dimension parameter value for the gth generation of the ith individual.

The steps to achieve this are as follows:

Step 1: Mutate each individual: select three individuals randomly selected as a reference and calculate their difference scores (obtained by subtracting two of them). Then, add these difference scores to the current individual to produce a new individual.

Step 2: Perform a crossover operation for each mutated individual with a certain probability to produce a new individual.

Step 3: Select the resulting new individuals through a tournament: compare the fitness values of the new individuals with those of the original individuals and retain the better-adapted individuals as the next generation of the population.

Step 4: Repeat the above steps until the set number of iterations of differential evolution is reached and stop.

#### 3.3.3. Hybrid Algorithm

(1) Cubic B-spline smoothing path

Aiming at addressing the problems that turns in raster maps produce spikes [32] and that the planned paths do not satisfy the kinematic constraints of the articulated steering tractor, this paper adopts cubic B-spline curves to smooth the paths, which eliminates unnecessary details and sharp edges in the original paths to satisfy the constraints of the curvature continuum and the turning radius.

The B-spline curve equation is:

(14) $$p\left(u\right)=\overset{n}{\underset{i=0}{\sum }}d_{i}N_{i,p}\left(u\right)$$

where $d_{i}\left(i=0,1\ldots n\right)$ is the control vertex and $N_{i,p}\left(i=0,1\ldots n\right)$ is p times the canonical B-spline basis function with the highest number of p.

The basis function is a pth order segmented polynomial determined by a sequence $U:\;u_{0}\leq u_{1}\leq \ldots \leq u_{n+p+1}$ of nondecreasing parameters u called node vectors. The B-spline curve basis function uses the Cox-deBoor recursive formula [32] as follows:

(15) $$\left\{\begin{array}{c} N_{i,\;0}\left(u\right)=\left\{\begin{array}{c} 1\;if\;u_{i}\leq u<u_{i+1}\\ 0\;otherwise \end{array}\right.\\ N_{i,\;p}\left(u\right)=\frac{u-u_{i}}{u_{i+p}-u_{i}}N_{i,\;p-1}\left(u\right)+\frac{u_{i+p+1}-u}{u_{i+p+1}-u_{i+1}}N_{i-1,p-1}\left(u\right)\\ define\;\frac{0}{0}=0 \end{array}\right.$$

where i is the node serial number and p is the order of the basis function. There are n+1 control vertices.

The articulated steering tractor operation model must be analyzed in order to ensure that the planned path is practical to avoid any tractor steering problems and to ensure the curvature of any point in the planned path to meet the constraints is as follows:

(16) $$\kappa_{i}\leq \frac{1}{R_{min}}$$

where $\kappa_{i}$ is the curvature of point $P_{i}$ calculated according to Equation (10) and $R_{min}$ is the minimum turning radius of the articulated steering tractor chassis.

(2) Algorithmic hybrid

The optimization goal of the improved A* algorithm is single, so it is difficult to meet the multi-objective requirements of symmetrical, uniform, and low power consumption of the bending steering tractor in the orchard, such as spraying, weeding, fertilization, and so on. The initial population of the improved genetic algorithm is randomly generated based on boundary constraints, so it is difficult to generate passable solutions quickly. In order to solve the above problems, this paper proposes the AGADE hybrid algorithm, which takes the solution generated by the improved A* as the initial population of the genetic algorithm. The individual of the initial population of the genetic algorithm is a better solution at the beginning, which can accelerate the convergence speed of the algorithm. Then, the differential evolution genetic algorithm is used to cross-mutate them, calculate the fitness function, select the better individual through the tournament, and iterate the cycle to obtain the optimal path. Since the map in this paper is a raster map, the path turns produce spikes and the path does not satisfy the articulated steering tractor turning radius constraints, the optimal path obtained is constrained and smoothed by using a cubic b-spline curve to satisfy the demand.

**Figure 7:**
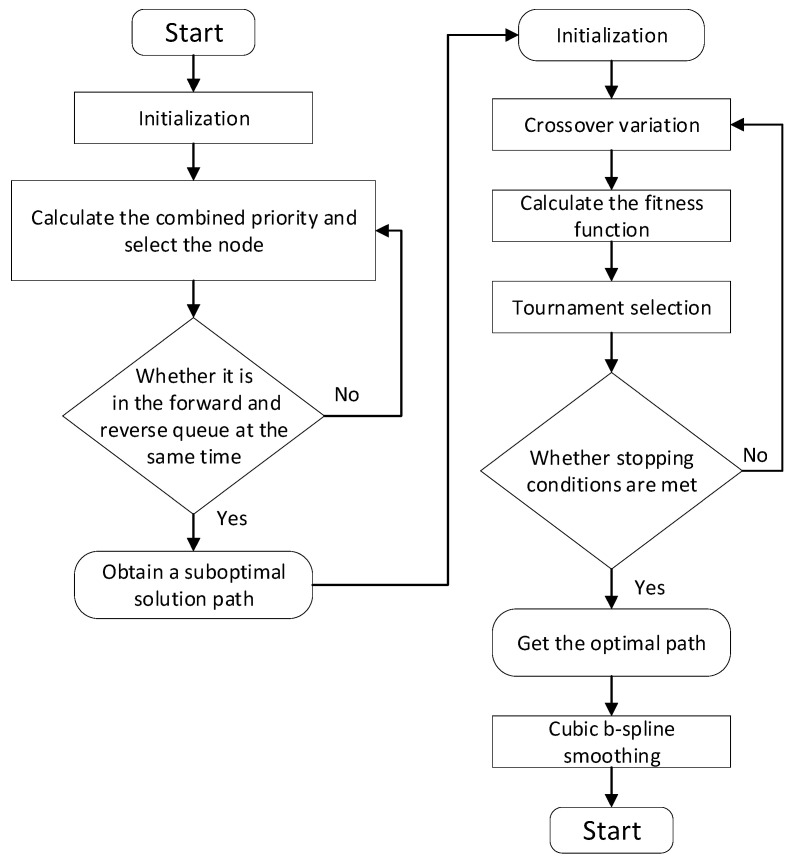
Flowchart of the AGADE hybrid algorithm.

The flow of the AGADE hybrid algorithm is shown in Figure 7, and the specific steps are as follows:

Step 1: Improving the integrated priority function, the bidirectional A* algorithm starts searching for suboptimal paths.

Step 2: The forward and backward search queues of the bidirectional A* algorithm meet and the bidirectional A* algorithm terminates to find a path from the start to the end.

Step 3: To initialize the population, the path obtained in Step 2 is used as the initial population for the improved genetic algorithm.

Step 4: The population is cross-mutated by differential evolution, and the fitness function is calculated by adding a path tree row center offset factor, a smoothing factor, and a safety factor to the fitness function, and the tournament method selects the next generation of the population.

Step 5: Step 4 is looped until the maximum number of iterations is reached, the algorithm is terminated, and the path is returned.

Step 6: The path returned in Step 5 is smoothed and constrained using a cubic b-spline curve algorithm.

Step 7: The final path and iteration convergence curves are plotted.

## 4. Simulation and Analysis

The simulation environment is as follows: the compilation environment is MATLAB 2022b, the computer system environment is Windows 11, and the running memory is 8 GB.

The path planning of the AGADE hybrid algorithm and the traditional GA algorithm are simulated, respectively. The AGADE hybrid algorithm takes the suboptimal solution produced by the A* algorithm as the initial population of the improved genetic algorithm, and the convergence speed should be improved in theory. The path trajectory in the AGADE hybrid algorithm for multi-objective optimization meets the needs of orchard operation in theory. Therefore, this section focuses on comparing and analyzing the convergence speed and trajectory effect of the AGADE hybrid algorithm and the traditional GA algorithm. The specific algorithm parameters are set as shown in Table 1.

**Figure 8:**
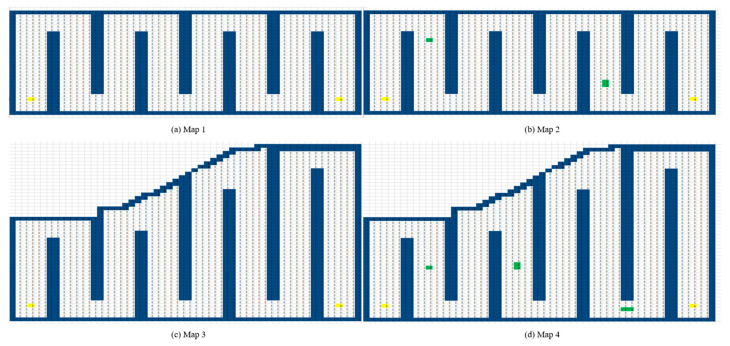
Map images. The yellow squares represent the starting and target points of the tractor, and the green squares represent obstacles.

According to whether the orchard scene is regular and whether there are obstacle points in the middle of the tree rows, four different complexity maps are set up as shown in Figure 8 for comparative analysis. Map 1 is a regular map with no obstacles in the tree rows, Map 2 is a regular map with obstacles in the tree rows, Map 3 is an irregular map with no obstacles in the tree rows, and Map 4 is an irregular map with obstacles in the tree rows. It is assumed that the distance between the tree rows is greater than the lateral width of the tractor, and the obstacles are treated with expansion.

Table 2 shows the data table of the simulation analysis results of the proposed AGADE hybrid algorithm and the traditional GA algorithm in four different complexity maps. In terms of the number of iterative convergence the AGADE algorithm shows significant improvement over the GA algorithm in all four maps. In terms of the path length, the AGADE algorithm plans a larger path length compared with the GA algorithm.

**Figure 9:**
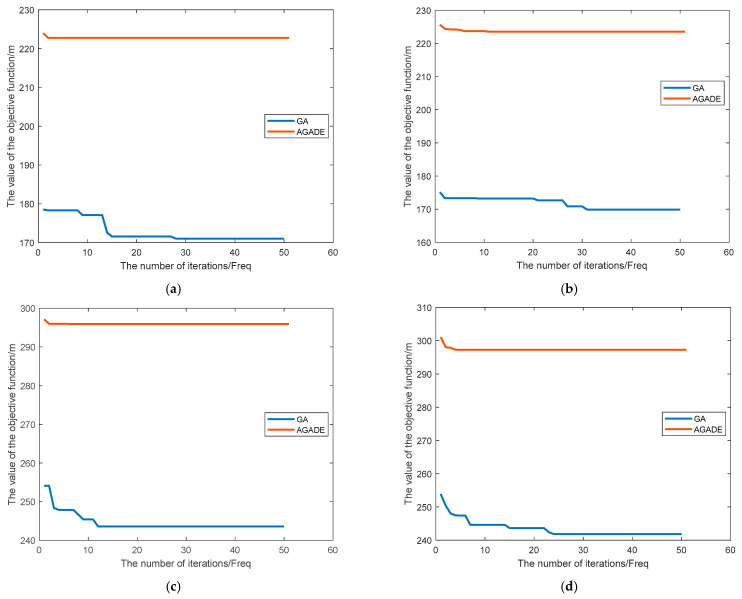
Comparison of target iteration convergence curves. (**a**) Map 1. (**b**) Map 2. (**c**) Map 3. (**d**) Map 4.

Figure 9 shows the variation curves of the number of convergence curves versus the objective function value for the AGADE hybrid algorithm and the GA algorithm for path planning on four different complexity maps. The number of convergence curves is related to the initial population generation method of the algorithms, in which the initial population of the AGADE hybrid algorithm is the suboptimal solution generated by the improved A* algorithm, while the traditional GA algorithm is randomly generated by relying on the boundary constraints. In Map 1, AGADE finds the shortest path length of 222.8 at the 2nd iteration and GA finds the shortest path length of 171.0 at the 28th iteration; AGADE iterations are reduced and the speed of convergence is improved by 92.8% compared with the GA algorithm. In Map 2, AGADE finds the shortest path length of 223.5 at the 11th iteration and GA finds the shortest path length of 169.9 at the 31st iteration; the AGADE hybrid algorithm has a reduced number of iterations and the convergence speed is increased by 64.5% compared with the GA algorithm. In Map 3, AGADE finds the shortest path length of 295.9 at iteration 6 and GA finds the shortest path length of 243.6 at iteration 12. The AGADE hybrid algorithm has a reduced number of iterations and the convergence speed is improved by 50.0% compared with the GA algorithm. In Map 4, AGADE finds the shortest path length of 297.2 at the 5th iteration and GA finds the shortest path length of 241.8 at the 24th iteration; the AGADE hybrid algorithm has a reduced number of iterations and the convergence speed is improved by 71.2% compared with the GA algorithm.

From the above results, it can be seen that the AGADE hybrid algorithm has stronger search ability, which is mainly due to the introduction of differential evolution measures in the AGADE hybrid algorithm; the speed of convergence is greatly improved, which is mainly due to the fact that the AGADE hybrid algorithm adopts the paths obtained by the bidirectional search of the improved A* algorithm as the initial population for iterative optimization, and the initial population of the genetic algorithm is the suboptimal solution generated by the improved A* algorithm. The initial population of the genetic algorithm is the suboptimal solution generated by the improved A* algorithm, which reduces the difficulty of obtaining the optimal path and improves the convergence speed compared with the traditional genetic algorithm that generates the initial path randomly. The reason that the path lengths of AGADE are increased in all four different complexity maps compared with GA is that the optimization direction of AGADE is not only generating the shortest paths but also considering whether the paths are located in the middle of the tree rows, smoothing, and other orchard operation requirements.

**Figure 10:**
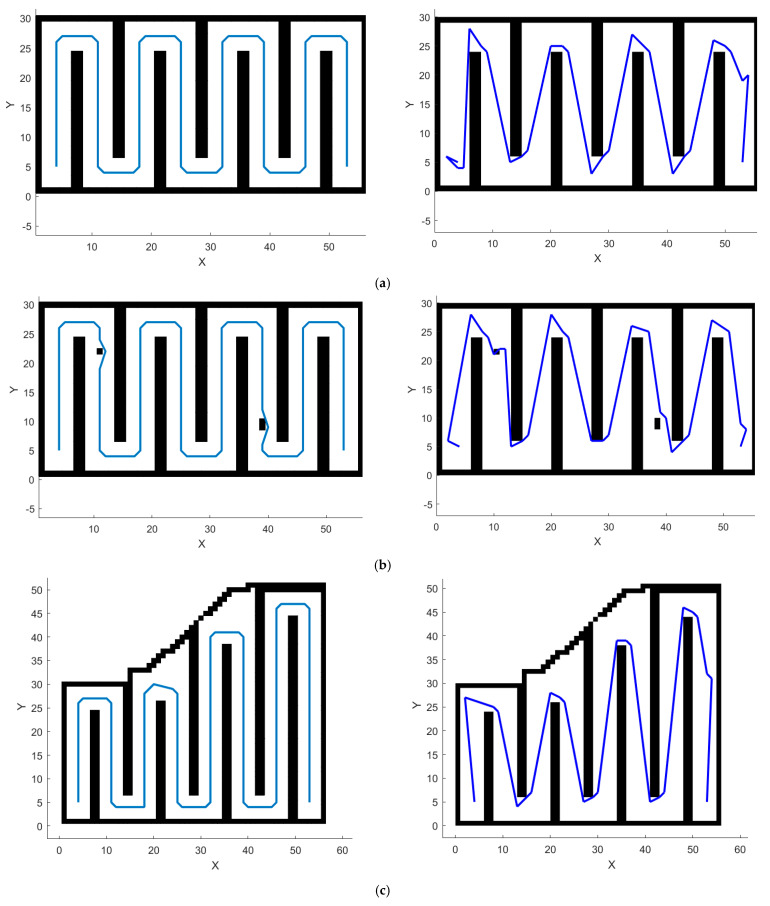

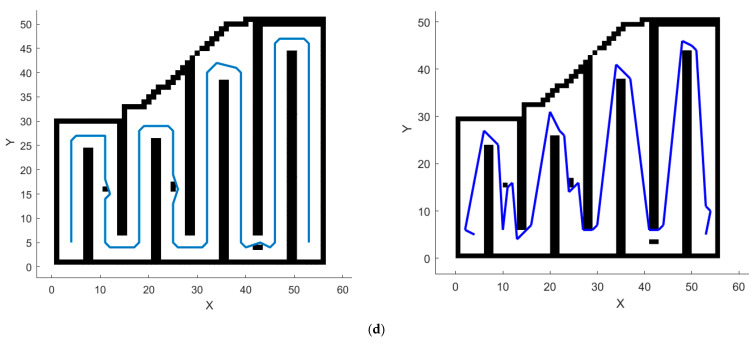
Comparison of path trajectories between the AGADE and GA algorithms in four maps. (**a**) Comparison of path trajectories in Map 1. (**b**) Comparison of path trajectories in Map 2. (**c**) Comparison of path trajectories in Map 3. (**d**) Comparison of path trajectories in Map 4.

Figure 10 shows the comparison of path trajectories between AGADE and GA in four different complexity maps. It can be seen that GA is prone to problems such as local optimization, path confusion, and the inability to avoid obstacles in the tree rows when performing path planning, which results in the planned paths not being able to be used in practice. At the same time, the path planned by AGADE is located in the center of the tree rows, can avoid obstacles in the tree rows, and has fewer turns, which can meet the operational needs of the articulated steering tractor in the orchard environment.

**Figure 11:**
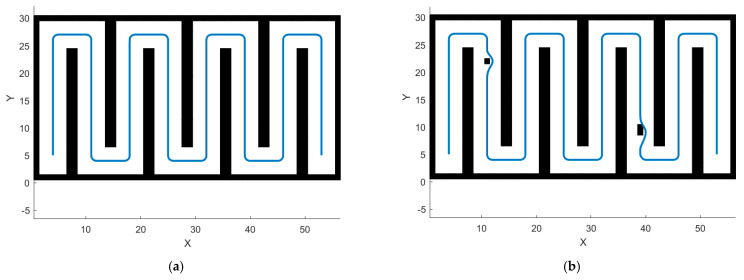

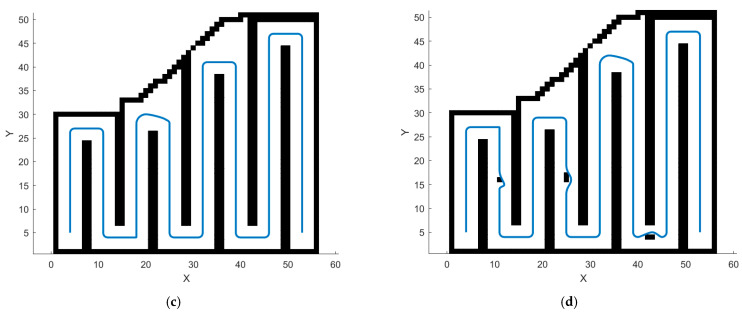
AGADE algorithm path trajectories after smoothing on four maps. (**a**) Smooth trajectory in Map 1. (**b**) Smooth trajectory in Map 2. (**c**) Smooth trajectory in Map 3. (**d**) Smooth trajectory in Map 4.

Figure 11 shows the path trajectories after the AGADE hybrid algorithm is smoothed on the four respective maps. Compared with scenarios before smoothing, problems such as spikes at the turns caused by the use of grid maps are solved, and the actual traveling is smoother to meet the demand of the actual orchard operation of the articulated steering tractor.

## 5. Conclusions

In this paper, a global path-planning algorithm for an articulated steering tractor in the orchard environment is investigated, an improved hybrid algorithm of bidirectional search A* and an improved differential evolution genetic algorithm (AGADE) is proposed, and simulation tests and analysis are carried out. General A* path planning only considers the shortest path, which is not in line with the demand of orchard operations, and genetic algorithms have problems such as local optimization and chaotic planning paths. This paper conducts the following research:

(1) Improved bidirectional A* algorithm: considers the effect of weights in the integrated priority function of the A* algorithm and changes the unidirectional search of the traditional A* algorithm to a bidirectional search, which reduces the number of search nodes and improves the search speed.
(2) Improved differential evolution genetic algorithm: the method of differential evolution is used to replace the cross mutation of the genetic algorithm, which improves the algorithm’s ability to explore the solution space and the algorithm’s global search ability, and avoids the problem of falling into the local optimal solution. The fitness function of the genetic algorithm is improved by adding the tree row center offset factor, the smoothing factor, and the safety coefficient, and the weighting method is used to transform the multi-objective optimization into single-objective realization.
(3) Improved bidirectional A* and improved differential evolution genetic hybrid algorithm: the path planned by the improved bidirectional search A* algorithm is used as the initial population of the differential evolution genetic algorithm, iterative optimization obtains the path trajectory curve, and the three-times B-spline curve is used to smooth the curve, obtaining the optimal path that is smooth and satisfies the constraints.

From the simulation comparison test and analysis, it can be seen that the improved bidirectional A* and the differential evolution genetic hybrid algorithm (AGADE) proposed in this paper has significantly improved the convergence speed over the genetic algorithm in complex environments, and the convergence speeds on four different complexity maps have been improved by 92.8%, 64.5%, 50.0%, and 71.2%, respectively. The path trajectory curves can meet the needs of the path located in the middle of the tree rows, the path is as short as possible, the path is smooth, and so on, so the articulated steering tractor meets the orchard operation requirements.

Deficiencies and prospects: The proposed path planning method has not been applied to real vehicles; to ensure that the articulated steering tractor can travel along the planned path, it is necessary to develop a path-tracking control method that conforms to the motion characteristics of the articulated steering tractor.

## Figures and Tables

**Table 1:** Comparison parameter list of the simulation test.

Basic Parameters	Data	Traditional Genetic Algorithm	Data	Differential Evolution Genetic Algorithm	Data
Initial population size	50	Multipoint crossover probability	0.9	Number of iterations of differential evolution	10
Maximum number of iterations	50	Single-point crossover probability	0.1		

**Table 2:** Comparison of the AGADE hybrid algorithm with GA.

Serial Number	Algorithms	Path Lengths	Number of Iterative Convergence Curves
1	AGADE	222.8	2
	GA	171.0	28
2	AGADE	223.5	11
	GA	169.9	31
3	AGADE	295.9	6
	GA	243.6	12
4	AGADE	297.2	5
	GA	241.8	24

## Data Availability

The original contributions presented in the study are included in the article, further inquiries can be directed to the corresponding author.

## References

[1] Liu B. (2020). Optimization design of the waist-steering mechanism based on the interior point method. J. Chin. Agric. Mech..

[2] Yue C., Huang J., Deng L. (2022). Research on improved ant colony algorithm in AGV path planning. Comput. Eng. Des..

[3] Shen Y., Liu Z., Liu H., Du W. (2023). Orchard Spray Robot Planning Algorithm Based on Multiple Constraints. Trans. Chin. Soc. Agric. Mach..

[4] Shi W., Ning N., Song C., Ning W. (2023). Path Planning of Mobile Robots Based on Ant Colony Algorithm and Artificial Potential Field Algorithm. Trans. Chin. Soc. Agric. Mach..

[5] Liu H., Zhang Y. (2022). ASL-DWA: An Improved A-Star Algorithm for Indoor Cleaning Robots. IEEE Access.

[6] Zhang J., Wu J., Shen X., Li Y. (2021). Autonomous land vehicle path planning algorithm based on improved heuristic function of A-Star. Int. J. Adv. Robot. Syst..

[7] Huang C., Ji F., Liu Y., Li H., Liu X. (2017). Smooth Path Planning Method Based on Dynamic Feedback A* Ant Colony Algorithm. Trans. Chin. Soc. Agric. Mach..

[8] Wang H., Zhao X., Yuan X. (2022). Robot path planning based on improved adaptive Genetic Algorithm. Electron. Opt. Control.

[9] Ab Wahab M.N., Nazir A., Khalil A., Ho W.J., Akbar M.F., Noor M.H.M., Mohamed A.S.A. (2024). Improved genetic algorithm for mobile robot path planning in static environments. Expert Syst. Appl..

[10] Liang C. (2023). Application of ant Colony Genetic Algorithm to Pathplanning of Substation Inspection Robot. Master’s Thesis.

[11] Shi K., Huang L., Jiang D., Sun Y., Tong X., Xie Y., Fang Z. (2022). Path planning optimization of intelligent vehicle based on improved genetic and ant colony hybrid algorithm. Front. Bioeng. Biotechnol..

[12] Li Y., Zhao J., Chen Z., Xiong G., Liu S. (2023). A robot path planning method based on improved genetic algorithm and improved dynamic window approach. Sustainability.

[13] Wang Q., He J., Lu C., Wang C., Lin H., Yang H., Li H., Wu Z. (2023). Modelling and control methods in path tracking control for autonomous agricultural vehicles: A review of state of the art and challenges. Appl. Sci..

[14] Viadero-Monasterio F., Nguyen A.T., Lauber J., Boada M.J.L., Boada B.L. (2023). Event-triggered robust path tracking control considering roll stability under network-induced delays for autonomous vehicles. IEEE Trans. Intell. Transp. Syst..

[15] He J., Hu L., Wang P., Liu Y., Man Z., Tu T., Yang L., Li Y., Yi Y., Li W. (2022). Path tracking control method and performance test based on agricultural machinery pose correction. Comput. Electron. Agric..

[16] Chen J., Niao C., Zhu Z. (2005). Study on Automatic Guidance for Tractor on Grassland. Trans. Chin. Soc. Agric. Mach..

[17] Zhang Q., Zhou B., Zhao J., You Y., Wang D.C. (2022). An Adaptive Parametric Model Predictive Path Tracking Control Method for Articulated Steering Tractor.

[18] Liu W.L. (2023). Research on Multi-Robot Motion Planning for Nonholonomic Kinematic Constraint Mobile Robots. Master’s Thesis.

[19] Cao R., Zhang Z., Li S., Zhang M., Li H., Li M. (2021). Multi-machine Cooperation Global Path Planning Based on A-star Algorithm and Bezier Curve. Trans. Chin. Soc. Agric. Mach..

[20] Ye X., Zhong H., Deng K. (2022). A Path Planning Method of Indoor Navigation Based on lmproved A-Star Algorithm. Comput. Technol. Dev..

[21] Hu Y., Yang S. (2004). A knowledge based genetic algorithm for path planning of a mobile robot. Proceedings of the IEEE International Conference on Robotics and Automation, 2004. Proceedings. ICRA’04. 2004.

[22] Zhan W., Li Y. (2023). Summary of Related Research on Path Planning Based on lmproved Genetic Algorithm. Comput. Digit. Eng..

[23] Kumar R., Kumar M. (2010). Exploring genetic algorithm for shortest path optimization in data networks. Glob. J. Comput. Sci. Technol..

[24] Liu X. (2024). Research on Logistics Delivery Path Optimization Based on Improved Genetic Algorithm. Master’s Thesis.

[25] Qiu L. (2024). Research on Vehicle Routing Problem Based on Improved Genetic Algorithm. Proceedings of the 2019 Chinese Automation Congress (CAC).

[26] Wang H., Yin P., Zheng W., Wang H., Zuo J. (2020). Mobile Robot Path Planning Based on lmproved A* Algorithm and Dynamic Window Method. Robot.

[27] Li J. (2024). Research on Path Planning of Mobile RobotBased on Improved A * Algorithm. Master’s Thesis.

[28] Zhang H., Tao Y., Zhu W. (2023). Global Path Planning of Unmanned Surface Vehicle Based on Improved A-Star Algorithm. Sensors.

[29] Wang J., Wang X., Tian Q., Sun A., Zhang X., Yuan L. (2021). Mobile Robot Path Planning Based on lmproved Fuzzy Adaptive Genetic Algorithm. Mach. Tool Hydraul..

[30] Li Y., Huang Z., Xie Y. (2020). Path planning of mobile robot based on improved genetic algorithm. Proceedings of the 2020 3rd International Conference on Electron Device and Mechanical Engineering (ICEDME).

[31] Zhao P., Ding X., Cheng T., Mo X. (2024). Improved Genetic Algorithm for UUV Path Planning Based on Differential Evolution Algorithm. Autom. Appl..

[32] Xing N., Di H., Yin W., Han Y., Zhou Y. (2023). Path planning for agents based on adaptive polymorphic ant colony optimization. J. Beijing Univ. Aeronaut. Astronaut..

